# Passage of influenza A/H3N2 viruses in human airway cells removes artefactual variants associated with neuraminidase-mediated binding

**DOI:** 10.1099/jgv.0.001348

**Published:** 2019-11-08

**Authors:** Jonathan C. Brown, Wendy S. Barclay, Monica Galiano, Ruth Harvey

**Affiliations:** ^1^​ National Institute for Biological Standards and Control, Potters Bar, UK; ^2^​ Department of Infectious Disease, Imperial College, London, UK; ^3^​ Public Health England, London, UK; ^†^​ Present address: WHO Collaborating Centre for Reference and Research on Influenza, Francis Crick Institute, London, UK

**Keywords:** influenza, H3N2, neuraminidase, vaccine, serology, haemagglutination

## Abstract

Serological assays with modern influenza A/H3N2 viruses have become problematic due to the progressive reduction in the ability of viruses of this subtype to bind and agglutinate red blood cells (RBCs). This is due to reduced ability of the viral haemagglutinin (HA) glycoprotein to bind to the sialic acid-containing receptors presented by these cells. Additionally, as a result of reduced HA-mediated binding in cell culture, modern A/H3N2 viruses often acquire compensatory mutations during propagation that enable binding of cellular receptors through their neuraminidase (NA) surface protein. Viruses that have acquired this NA-mediated binding agglutinate RBCs through their NA, confusing the results of serological assays designed to assess HA antigenicity. Here we confirm with a large dataset that the acquisition of mutations that confer NA binding of RBCs is a culture artefact, and demonstrate that modern A/H3N2 isolates with acquired NA-binding mutations revert to a clinical-like NA sequence after a single passage in human airway epithelial (HAE) cells.

## Introduction

Influenza A/H3N2 viruses have circulated in the human population since the Hong Kong pandemic of 1968 and currently co-circulate alongside influenza A/H1N1 viruses descended from the virus responsible for the 2009 pandemic. The seasonal influenza vaccine contains antigenic components from these two subtypes of influenza A, as well as from one or both of the currently circulating lineages of influenza B. Due to the rapid genetic and antigenic evolution of influenza, global surveillance of circulating viruses is continuous and prior to each flu season influenza viruses isolated from human infections worldwide are assessed to see if they have become antigenically distinct from the strains included in the previous season’s vaccine. While routine sequencing of influenza haemagglutinin (HA) glycoproteins can help to predict antigenic properties, serological assays are essential to demonstrate whether genetic changes confer enough antigenic drift to necessitate a vaccine strain update. The core assay used to make this assessment is the haemagglutination inhibition (HAI) assay, which determines the specificity of antiserum by measuring its ability to block the interaction between a virus and sialic acid-containing receptors on the surface of red blood cells (RBCs). However, the current use of this assay for the A/H3N2 viruses is complicated by the accumulation of mutations in the HA globular head of these viruses since 1968, which have altered the receptor-binding properties of these viruses and led to a reduction in their ability to bind and agglutinate RBCs. Receptor binding changes have also resulted in reduced isolation rates of A/H3N2 viruses in the Madin–Darby canine kidney (MDCK) cell line traditionally used to propagate influenza viruses [1, 2], reducing the pool of viruses that can be assessed at all and further compromising strain selection. Alongside these changes in HA-mediated binding, the phenomenon of neuraminidase (NA)-mediated binding by acquisition of a D151G mutation in the NA of passaged A/H3N2 isolates has also been observed [3]. G151 receptor-binding variants show reduced sialidase activity [4] and rather than reaching fixation in a viral population, co-exist alongside the D151 genotype, which codes for an enzymatically active NA [5]. In MDCK-SIAT cell culture, a viral population with a mixture of D151 and G151 genotypes is fitter than either variant in isolation, but only when paired with a modern H3 HA with low receptor avidity [5]. Using next-generation sequencing (NGS), G151 variants have recently been shown to be absent from a small panel of nine circulating A/H3N2 viruses collected between 2013 and 2015, which subsequently acquired these variants upon MDCK cell passage [6]. This supports the conclusions of others that NA-mediated binding is a cell culture artefact. Here we analysed a larger set of clinical samples collected in the 2014–15 season to confirm the absence of genetic variants associated with NA-mediated binding. We also reveal that a single passage in human airway epithelial (HAE) cells of isolates bearing genetic variants associated with NA-mediated binding can revert them back to a clinical genotype.

## Results

### NA-mediated agglutination of guinea pig RBCs is a result of a D151G mutation in the NA of A/Sydney/71/2014

**Fig. 1.**
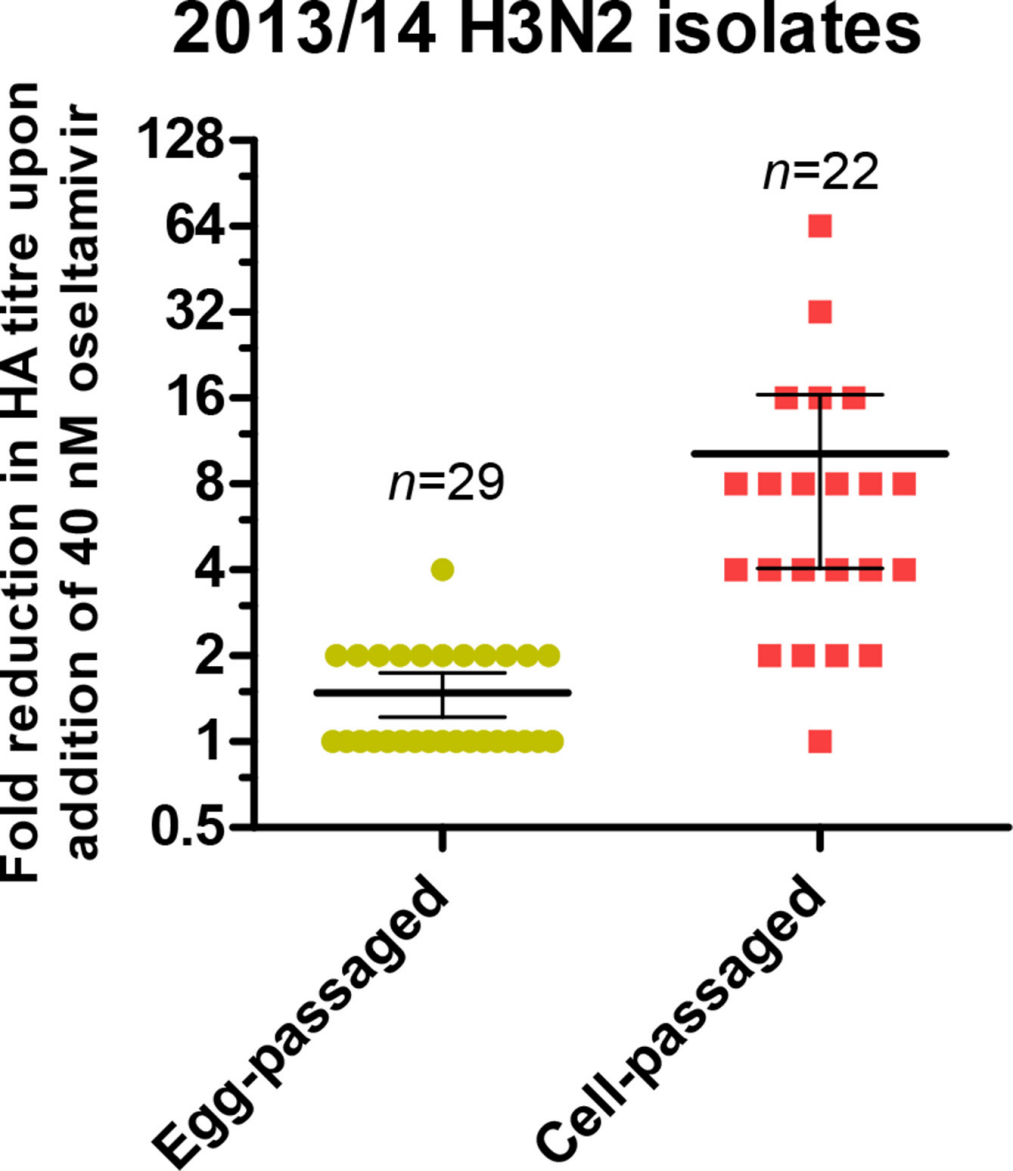
Reduction in the titre of egg- and cell-passaged A/H3N2 isolates from 2013–14 in the presence and absence of oseltamivir. The fold reduction in titre in the presence of 40 nM oseltamivir was recorded. The mean fold reductions was significantly different between egg-passaged and cell-passaged viruses (unpaired *t*-test, *P*=0.0221).

To investigate the phenomenon of NA-mediated agglutination of RBCs, A/H3N2 isolates that had been egg-passaged (*n*=29) or passaged in MDCK and/or MDCK-SIAT1 cells (*n*=22) were tested for the sensitivity of their HA titres to the presence of 40 nM oseltamivir (Fig. 1). Cell-passaged isolates showed significantly higher sensitivity than egg passaged strains, for example cell-passaged influenza A/Sydney/71/2014 (hereafter called Sydney/2014) demonstrated the greatest drop in HA titre from 128 to 2 in the presence of oseltamivir. Sanger sequencing revealed the presence of a mixed GAT/GGT genotype at the codon for residue 151 of the NA gene in Sydney/2014, which was quantified by NGS as 78 % GAT and 22 % GGT, resulting in 78 % aspartic acid (D) and 22 % glycine (G) amino acids at this position. Variants at residue 151 have previously been shown to alter the activity of NA, with the G151 resulting in a sialic acid-binding, sialidase-inactive phenotype [3, 4]. We generated variants of Sydney/2014 with either a D151 or G151 NA together with the HA of Sydney/2014 and the six remaining genes of A/Puerto Rico/8/34 (PR8) by reverse genetics. The G151 virus was generated and propagated by the addition of exogenous bacterial NA. We tested the sensitivity of the HA titres of the RG viruses to oseltamivir. Sydney/2014 with purely NA D151 showed no drop in titre in the presence of oseltamivir, while a purely NA G151 virus demonstrated an HA titre of 64, which was entirely abrogated in the presence of oseltamivir, demonstrating that this mutation conferred NA-mediated binding (Table 1).

**Table 1.** NA-mediated binding of Sydney/2014 is conferred by a D151G mutation in NA. RG viruses were generated with D151 and G151 NA and their titres recorded in the presence and absence of 40 nM oseltamivir. The HA titre of Sydney/2014 NA D151 was unchanged, while the HA titre of Sydney/2014 NA G151 was entirely abrogated by oseltamivir. Cell-passaged Sydney/2014 isolate (top) demonstrated an intermediate phenotype with a fourfold reduction in HA titre in the presence of oseltamivir owing to the minor (22 %) NA G151 population, determined by NGS

	Amino acid identity at NA151		Haemagglutination assay titre	
**A/Sydney/71/2014 virus type**	**D151**	**G151**	−**Oseltamivir**	**+Oseltamivir**
Cell-passaged clinical isolate	78 %	22 %	64	16
NA D151 RG virus	100 %	0 %	8	8
NA G151 RG virus	0 %	100 %	64	0

### Recent clinical A/H3N2 isolates do not contain the minor NA variants associated with NA-mediated binding

The appearance of NA variants in A/H3N2 viruses that mediate the agglutination of RBCs has implications for carrying out and interpreting the results of serological assays, but these variants may not be of clinical significance. To investigate whether such variants are found in circulating viruses, NGS data from 251 clinical A/H3N2 samples collected during the 2014–15 influenza season in the UK were assessed for the presence of NA variants at codon 151. The presence of variants was also assessed at codon 148. A T148I recombinant NA has demonstrated reduced catalytic activity and this mutation, along with T148K, can arise in MDCK cell-passaged isolates. These mutations have been shown to correlate with receptor-binding properties in haemagglutination assays [3, 7, 8]. Using a NGS strategy with a 1 % frequency cutoff for the detection of minority variants, none of the 251 clinical samples contained minor genetic variants that would result in amino acid substitutions at either residue, demonstrating that these variants are absent from circulating viruses and arise as a result of cell passage.

### Variants at residues 148 and 151 of NA arise more readily in MDCK cells

**Fig. 2.**
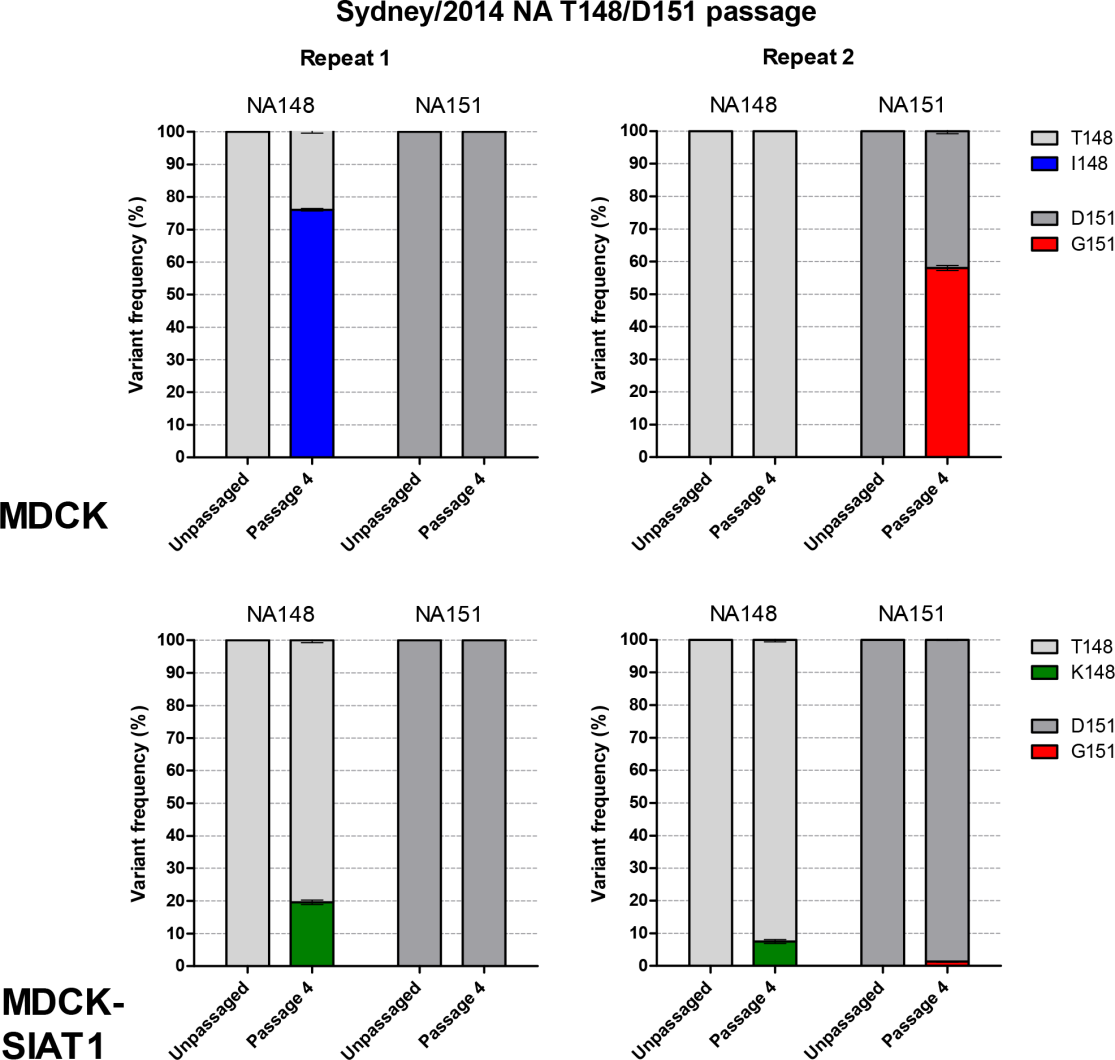
Selection of NA 148 and 151 variants after passage of Sydney/2014 NA D151 variant (RG353) in MDCK and MDCK-SIAT1 cell lines. RG353 was passaged four times at 0.01 p.f.u./cell in both cell lines in duplicate and the harvests from the fourth passage were sequenced by NGS. RT-PCR of vRNA was performed in triplicate and only SNPs observed above 1 % in all three repeats were reported. The mean of three replicates with the sem is shown.

To demonstrate *de novo* selection of NA variants during propagation in cell culture, the RG variant of Sydney/2014 with the NA T148/D151 (clinical-like) sequence was serially passaged four times at 0.01 p.f.u./cell in parallel in MDCK and MDCK-SIAT1 cells. Inocula were added to duplicate flasks and then serially passaged in parallel. NGS of virus harvests from the fourth passage showed that MDCK cells selected for NA-mediated binding mutations in both replicates. Interestingly, a different mutation arose in the virus in each of the parallel passages. In one replicate, T148I reached 76 % but no mutations were detected at 151, whereas in the second replicate D151G reached 67 % with no mutations detected at 148 (Fig. 2). Selection of variants still occurred in MDCK-SIAT1 cells, but to a lesser extent (Fig. 2). T148K reached 20 % in one harvest and in the other T148K reached 8 % with an accompanying D151G variant at 1 %. Passaged viruses did not acquire any fixed mutations in HA or NA. No minor variants were detected in the HA receptor-binding site (RBS) or at positions other than 148 or 151 in the NA catalytic site.

### Historical H150R mutation was not a prerequisite for the acquisition of NA 151 variants

**Fig. 3.**
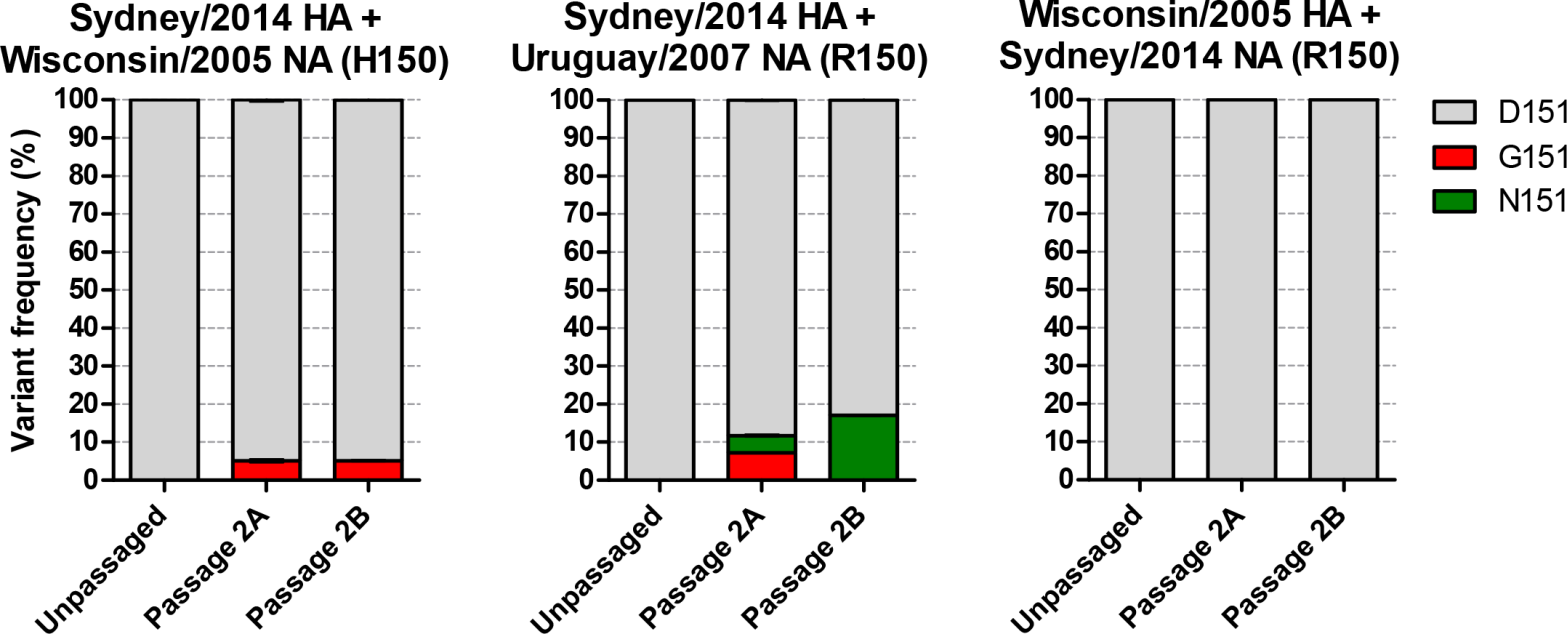
Selection of NA 151 variants in viruses bearing an H150 or R150 NA paired with an older or more modern HA. RG viruses were generated and passaged twice in duplicate at 0.01p.f.u./cell in MDCK-SIAT1 cell before sequencing by NGS. D151G/N variants were selected in both viruses with the H150 NA and R150 NA. The older Wisconsin/2005 HA was unable to drive selection of NA 151 variants in the Sydney/2014 NA. No minor variants were detected at 148 or 150 of NA.

It was shown recently [9] that an H150R mutation in the NA of A/H3N2 viruses was responsible for the NA-mediated agglutination of turkey RBCs. This mutation became fixed in the majority of circulating viruses around 2008. We were interested to see whether this was a prerequisite for the acquisition of NA 148/151 mutations that mediate haemagglutination in the guinea pig RBC assays that are now routinely carried out. Viruses were generated with the internal genes of PR8, the HA of Sydney/2014 and the H150 NA of A/Wisconsin/67/2005 (Wisconsin/2005) or the R150 NA of A/Uruguay/716/2007 (Uruguay/2007) and passaged twice in duplicate in MDCK-SIAT1 cells before sequencing by NGS (Fig. 3). Passage of the virus with the current R150 NA selected for a mixture of D151G/N variants totalling 12 % in one replicate with D151N at 17 % in the other. Interestingly, passage of the virus with the older H150 NA was still able to select for D151G variants to 5 % in both replicates, indicating that the H150R mutation was not required for acquisition of these variants. Parallel passage of a virus with the older HA of Wisconsin/2005 and the NA of Sydney/2014 with R150 showed that this older HA did not drive selection of 148/151 variants in the modern NA (Fig. 3). None of the viruses acquired variants at 148 or 150 of NA.

### NA G151 variants are not beneficial and are selected against in HAE cells

**Fig. 4.**
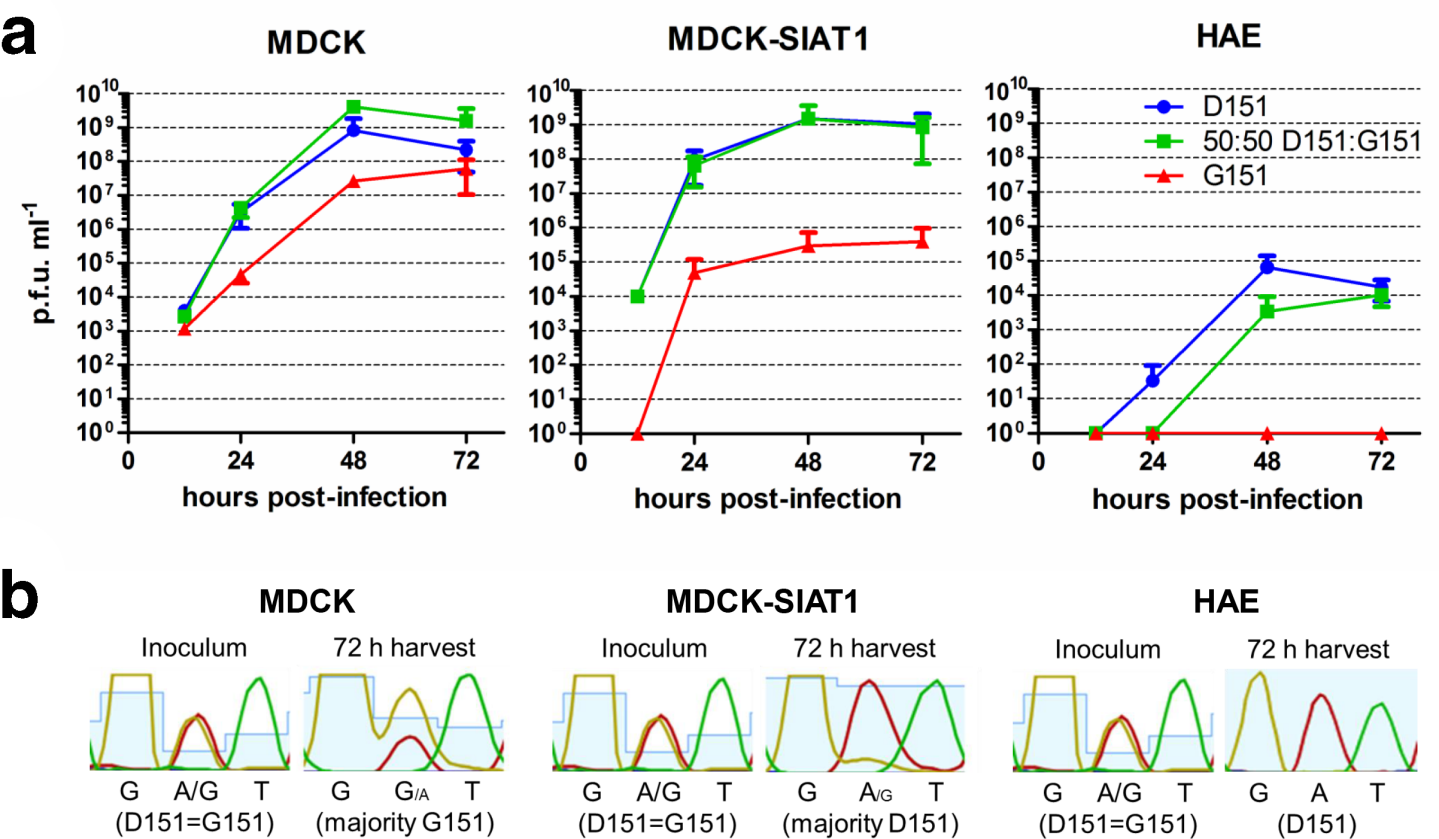
Complementation between viruses with D151 and G151 variant NAs occurs in MDCK but not MDCK-SIAT1 or HAE cells. (a) MDCK and MDCK-SIAT1 cell lines were infected in triplicate at 0.001 p.f.u./cell with Sydney/2014 bearing a D151 NA, G151 NA or a 50  :  50 mixture of the two viruses. HAE cells were infected at 0.02 p.f.u./cell. A mixture of variants grew better than either variant alone in MDCK cells but not in MDCK-SIAT1 cells. Pure NA D151 virus grew better than a 50  :  50 mixture of NA D151 and NA G151 viruses in HAE cells. (b) Genotype at NA residue 151 of virus harvests after infection with 50  :  50 D151:G151 mixture. Sanger sequencing was carried out on the inoculum and 72 h harvests. Virus inoculum showed equal GAT (D) and GGT (G) representation at NA residue 151. MDCK cells selected for a genotype weighted towards G151, whereas MDCK-SIAT1 cells selected for a predominantly D151 population. In HAE cells no G151 population was detected.

The absence of NA-mediated binding variants from circulating modern A/H3N2 viruses, and their selection in MDCK/MDCK-SIAT1 cells, suggests that such variants may arise due to the difference in sialic acid availability in different cell systems requiring alterations of the HA : NA balance for optimal virus fitness. We hypothesized that variants selected in MDCK and MDCK-SIAT1 cells would not be beneficial in primary HAE cells, which are more representative of the physiological environment where influenza virus replicates *in vivo*. MDCK, MDCK-SIAT1 and HAE cells were infected in triplicate at 0.001p.f.u./cell with RG Sydney/2014 variants with NA D151, NA G151 or a 50 : 50 mixture of these two variants and the viral titre was assessed by plaque assay at indicated time points (Fig. 4a) . In MDCK cells, at 48 h post-infection the mixture of variants had reached a higher titre (4.1×10^9^ p.f.u. ml^−1^) than the D151 variant alone (8.3×10^8^ p.f.u. ml^−1^). While this difference was not statistically significant due to the variability in titre across passage replicates, the data showed a clear trend for improved growth of the mixture. In MDCK-SIAT1 cells, a mixture of variants conferred no growth advantage over the D151 variant alone, with both reaching a titre of 1.5×10^9^ p.f.u. ml^−1^ by 48 h post-infection. The G151 variant reached a titre of only 3×10^5^ p.f.u. ml^−1^ in MDCK-SIAT1 cells. In HAE cells the D151 variant alone grew to 6.6×10^4^ p.f.u. ml^−1^, outperforming the mixture of variants (3.5×10^3^ p.f.u. ml^−1^) and the G151 variant whose titre was below the level of detection of the plaque assay. Seventy-two hour harvests from the mixed D151: G151 infections were sequenced and showed that whereas both MDCK and MDCK-SIAT1 cells retained mixtures of genotypes at codon 151, HAE cells selected for a population in which G151 variants were undetectable by Sanger sequencing (Fig. 4b).

### A single passage in HAE cells removes variants associated with NA-mediated binding

**Fig. 5.**
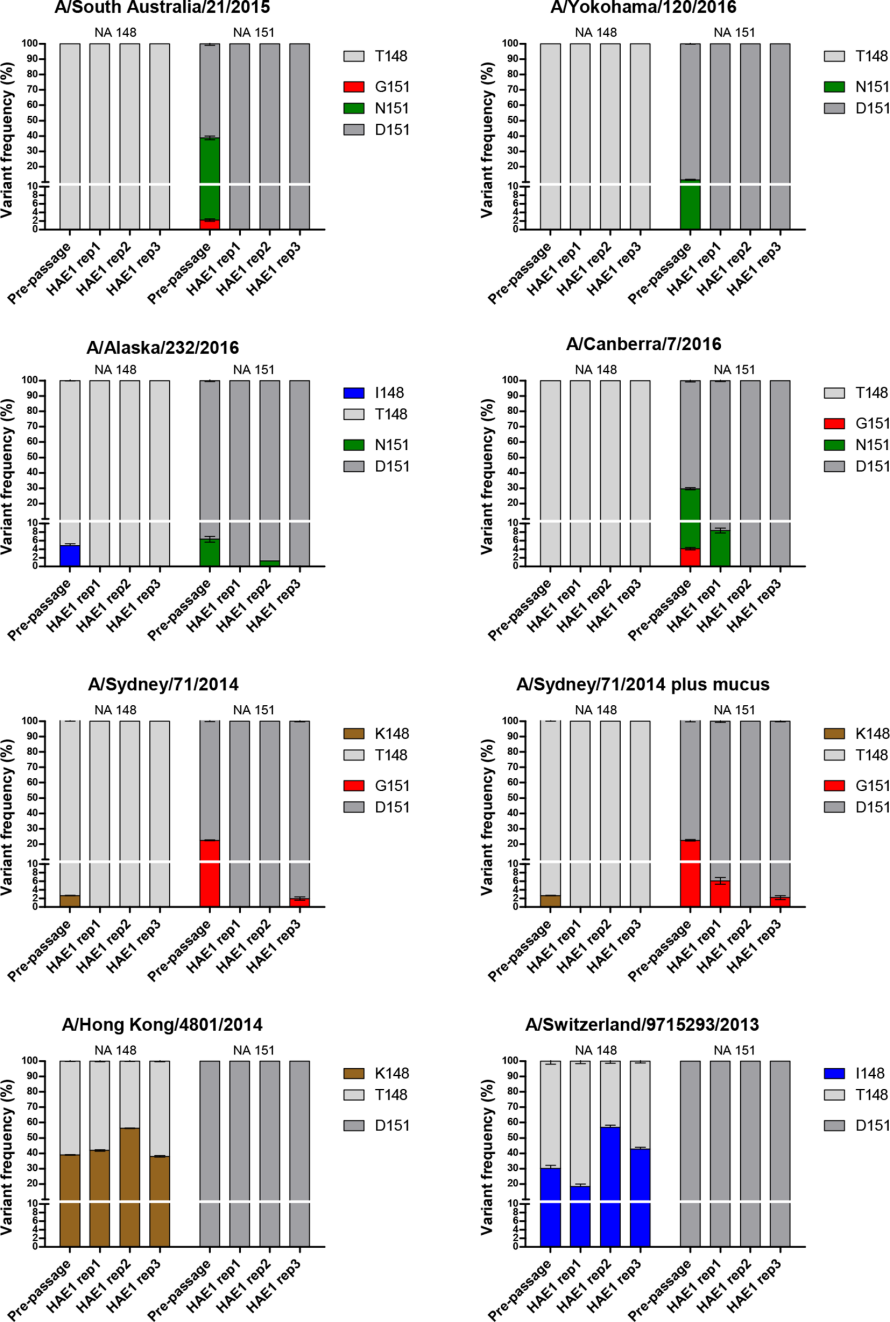
HAE cell selection at positions 148 and 151 of NA. Viruses with NA variants associated with NA-mediated binding were sequenced by NGS before and after a single passage in HAE cells at 0.02 p.f.u./cell. HAE cells were washed to remove mucus for all infections except those for A/Sydney/71/2014 plus mucus. Viruses were harvested 4 days post-infection. RT-PCR of vRNA was performed in triplicate with the mean and sem shown. South Australia/2015 and Yokohama/2015 were entirely cleared of NA-binding variants in all three replicates as a result of HAE cell passage.

**Fig. 6.**
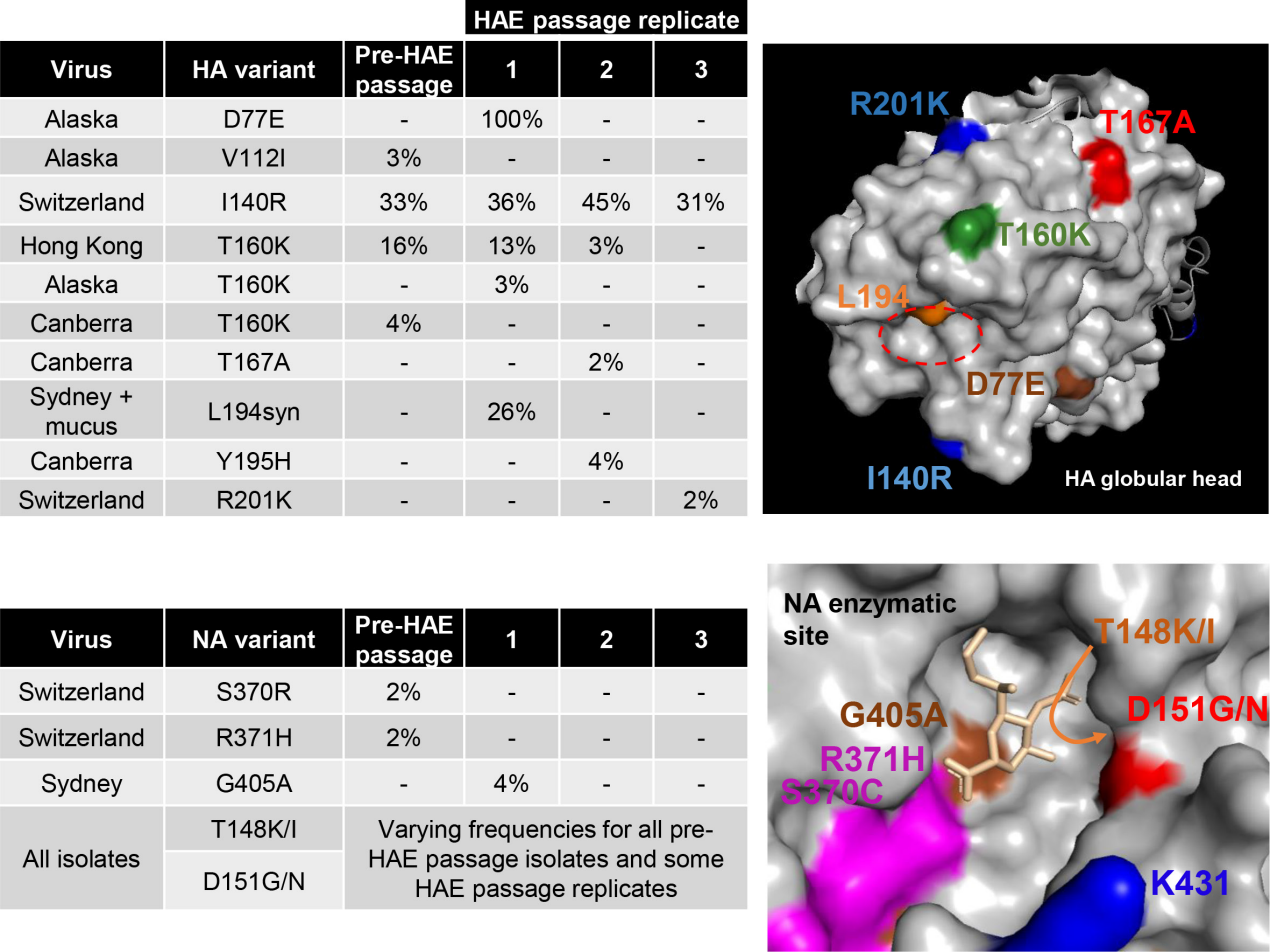
Position of HA and NA mutations acquired by panel of A/H3N2 viruses upon HAE cell passage. Top left: frequency of mutations acquired in HA globular head that may affect receptor binding and/or antigenicity. Percentage values are the average of three RT-PCR reactions from viral RNA samples. Top right: mutations mapped onto globular head of H3 structure (protein databank reference: 3ztj). The red dotted circle shows the location of the receptor-binding site. L194syn indicates a synonymous mutation. Bottom left: mutations in residues in and around the NA enzymatic pocket. Bottom right: mutations mapped onto the surface of human N2 globular head (protein databank reference: 4gzt). A close-up of an enzymatic pocket with sialic acid residue within is in gold.

Based on the observation that genotypic variants associated with NA-mediated binding were not replicated in HAE cultures, we hypothesized that passage through these cells could remove these variants from MDCK/MDCK-SIAT1-passaged viruses, allowing their subsequent use in serological assays. HA assays were conducted on A/H3N2 isolates from 2013-2016 (*n*=38) and a panel of seven isolates spanning subclades 3C.2a, 3C.2a1 and 3C.3a were chosen based on the sensitivity of their HA titres to oseltamivir (Table 2). The HA and NA genes of this panel of viruses were sequenced by NGS and each virus was used to infect HAE cells in triplicate at 0.001 p.f.u./cell. Washing to remove the mucus layer from HAE cells was carried out prior to infection for all samples except for a duplicate set of Sydney/2014 infections in which this wash step was omitted to compare any effect due to the presence of the mucus layer. PCR and plaque assays of 72 h harvests showed that for some of the input viruses, one or more of the replicate infections had failed to yield detectable virus. HAE cells were then reinfected at 0.02 p.f.u./cell and harvests taken after a further 4 days and sequenced by NGS. Pre-passage, all isolates contained variants at NA residues 148 and/or 151 (leftmost bars, Fig. 5). Post-HAE passage, in the majority of replicates (13/24) these laboratory-acquired mutations were undetectable (Fig. 5). Mutations elsewhere in the enzymatic site of NA or on the HA globular head, particularly in the RBS, may result in changes to the HA : NA functional balance. No non-synonymous mutations arose in the HA RBS of HAE-passaged isolates and the highest frequency mutation in the NA enzymatic site other than at 148/151 was G405A at 3.6 % in Sydney/2014 (Fig. 6).

**Table 2.** Panel of influenza A/H3N2 isolates with HA titres sensitive to oseltamivir. HA assays were conducted on *n*=38 cell-passaged isolates from 2013–162013-2016 in the presence or absence of 40 nM oseltamivir and those with the greatest titre reductions are recorded here. Passage level indicates the number of passages the isolate had undergone in MDCK and/or MDCK-SIAT1 cells prior to titration

				HA titre		
**Strain**	**Subtype**	**Clade**	**Passage level**	**Without oseltamivir**	**With oseltamivir**	**Fold drop in the presence of oseltamivir**
**A/Alaska/232/2016**	**H3N2**	**3C.2a1**	**SIAT5**	**16**	**0**	**total**
**A/Canberra/7/2016**	**H3N2**	**3C.2a**	**SIAT4**	**16**	**0**	**total**
**A/Hong Kong/4801/2014**	**H3N2**	**3C.2a**	**MDCK4SIAT3**	**16**	**0**	**total**
**A/South Australia/21/2015**	**H3N2**	**3C.2a**	**SIAT4**	**32**	**0**	**total**
**A/Switzerland/9715293/2013**	**H3N2**	**3C.3a**	**SIAT3MDCK2SIAT1**	**128**	**32**	**4**
**A/Sydney/71/2014**	**H3N2**	**3C.2a**	**MDCK4SIAT1**	**64**	**16**	**4**
**A/Yokohama/120/2016**	**H3N2**	**3C.2a**	**MDCK1SIAT3**	**16**	**2**	**8**

### Clinical genotype is maintained upon subsequent passage in MDCK-SIAT1 cells for some isolates

**Fig. 7.**
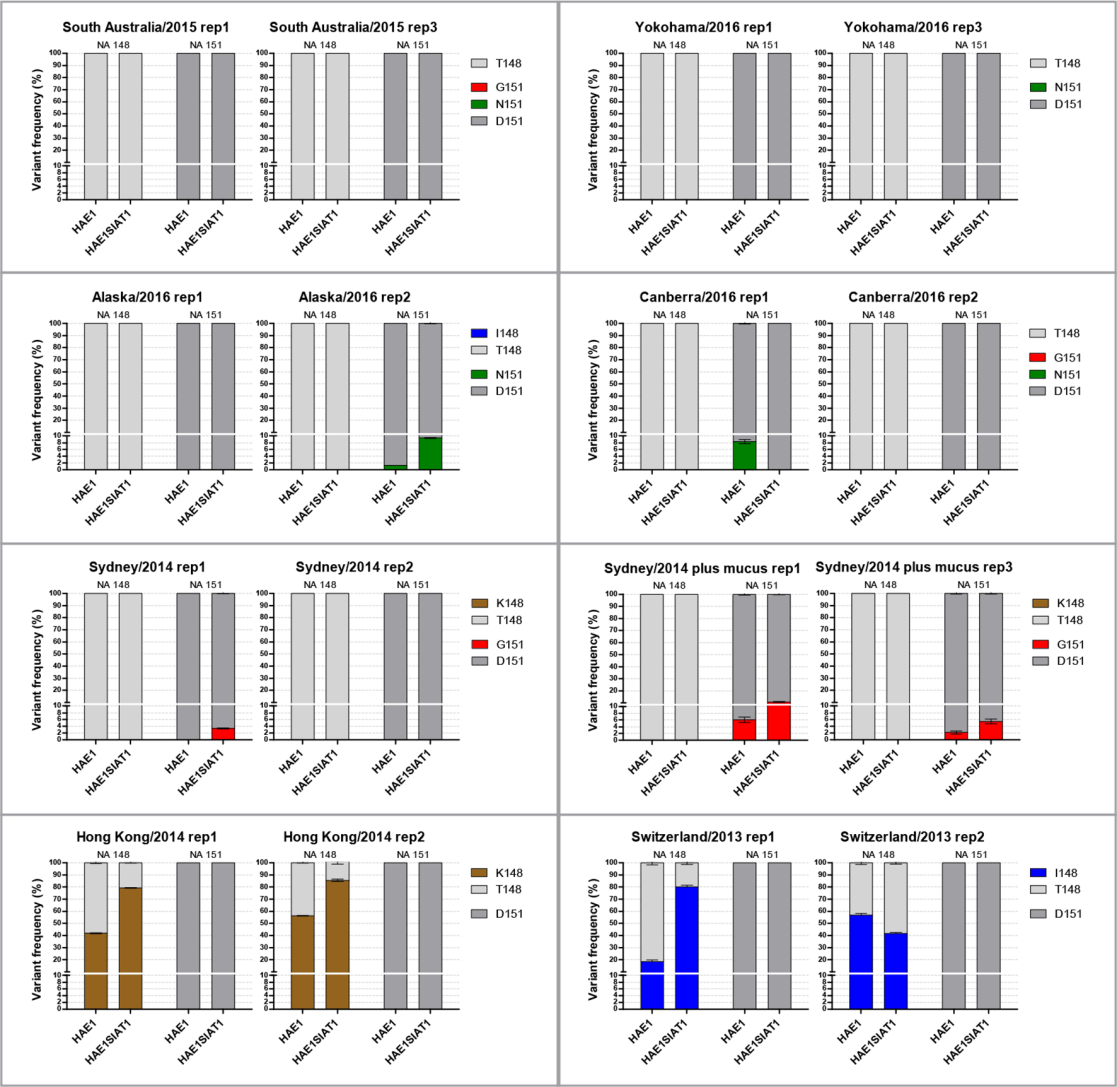
Frequency of NA 148 and NA 151 variants after passage in HAE cells and continued passage in MDCK-SIAT1 cells. Each chart gives variant frequencies at each passage for one virus. ‘rep1’ indicates the first HAE passage replicate for the given virus and the second bar for each chart gives the variant frequency after this HAE harvest was subsequently passaged in MDCK-SIAT1 cells. Isolates which were cleared of NA-binding variants by HAE passage remained clear through the subsequent MDCK-SIAT1 passage, except for Sydney/2014 rep 1, which spontaneously reacquired G151 variants.

The use of HAE cells to return passaged A/H3N2 isolates to a clinical sample-like genotype proved effective for the majority of viruses in the panel. However, HAE cells only yielded a small volume of virus harvest, which must be scaled up in order to make using these cells a viable method for the production of virus stocks that can be shared between laboratories and used for serological assays. Two of the HAE harvests from each starting isolate were used to infect MDCK-SIAT1 cells at 0.01 p.f.u./cell, harvested after 72 h and sequenced by NGS. Fig. 7 tracks the frequency of variants at NA 148 and NA 151 from pre-HAE passage, through the passage in HAE cells (HAE1) and through the subsequent passage of this material in MDCK-SIAT1 cells (HAE1SIAT1). Of the selected HAE cell harvests, 8 out of 16 (50 %) were free of NA variants. Of these eight NA-mediated binding-free isolates, seven still had undetectable levels of 148 and/or 151 NA variants after subsequent MDCK-SIAT1 cell passage.

## Discussion

This work demonstrates that NA-mediated binding of influenza A/H3N2 isolates in serological assays, which arises as an artefact of MDCK/MDCK-SIAT1 cell passage, may be removed by passage in HAE cells. MDCK cells are commonly used for the propagation of influenza viruses; however, poor growth, reduced isolation rates and the selection of adaptive mutations in the HA and NA surface proteins when recent A/H3N2 viruses are passaged in these cells prohibits their use [2, 10, 11]. The use of the MDCK-SIAT1 cell line, modified to express more of the α-2,6-linked sialic acid-containing receptors used by human-adapted influenza viruses, has helped to tackle these challenges [1, 2]. However, adaptive mutations may still be selected for in MDCK-SIAT1 cells and the infectivity of modern A/H3N2 viruses may be compromised when passaging at multiplicities of infection that prevent them from arising [8, 12].

Cell passage of A/H3N2 viruses can select for mutations in NA, such as D151G, which confer receptor-binding properties with a concomitant reduction in sialidase activity [3, 4]. Such artefactual NA variants were recently shown to be absent from a set of nine A/H3N2 clinical samples from 2013–15, which later selected for them in cell culture [6]. Analysis of NGS data for 251 A/H3N2 clinical samples from 2014–15 showed that NA-mediated binding variants were also absent from this larger sample set and supports the conclusion that such variants arise *de novo* during propagation in cell culture. While absent from circulating viruses, the presence of these variants in cell-passaged viruses necessitates the inclusion of oseltamivir in serological assays to remove the NA-mediated component of receptor binding.

Sydney/2014, a cell-passaged A/H3N2 virus from the recent 3C.2a subclade, exhibits the problematic phenotype of NA-mediated agglutination of RBCs and the generation of isogenic viruses at position 151 of NA demonstrated that this was caused by the D151G mutation (Table 1). Serial passage of the recombinant virus with the T148/D151 clinical sample-like NA sequence demonstrated selection towards a mixed genotype at 148 and/or 151, rather than fixation of either variant (Fig. 2) . Interestingly, in MDCK cells T148I and D151G rose separately to >50 % frequency in the viral population in the duplicate serial passages of this virus, demonstrating the stochastic nature by which *de novo* mutations arise and are then positively selected for to carry out the function of NA-mediated binding (Fig. 2). MDCK-SIAT1 cells selected for 148 and/or 151 variants to <20 % of the viral population (Fig. 2), supporting the idea of their use in maintaining the clinical sequence of A/H3N2 viruses more faithfully than MDCK cells, while highlighting that adaptations can still occur [8, 12].

Mögling *et al*. [9] identified that an H150R mutation hat fixed in A/H3N2 viruses around 2008 confers the ability of NA to agglutinate turkey RBCs. Sydney/2014 carries R150 but RG variants demonstrate that it is the identity of the amino acid at position 151 that confers the sensitivity of its HA titre to oseltamivir when using guinea pig RBCs (Table 1). As noted by Mögling *et al*. [9], RBCs from different species carry different sialic acid-terminating glycan structures to which HA and NA may bind. Over the years, a succession of RBCs from different species have been used to keep pace with the evolution of the receptor-binding properties of A/H3N2 viruses. A/H3N2 viruses first lost the ability to agglutinate chicken RBCs around 2001 and turkey RBCs around 2005, despite turkey cells presenting a greater number of the α-2,6-linked sialic acid-terminating receptors bound by HA [13, 14]. Current routine practice is to use guinea pig RBCs in serological assays for A/H3N2 viruses, although some recent isolates fail to bind even these RBCs (Lin *et al.*, 2017). Mögling *et al*. [9] demonstrated the ability of an R150 NA to mediate the haemagglutination of turkey RBCs when paired with a 2009 HA that haemagglutinates these cells poorly. Our findings suggest that for more modern A/H3N2 viruses it is minor variants at 148/151 acquired during cell culture passage that confer NA-mediated binding of guinea pig RBCs and that this confounds current serological assays. We have demonstrated that the H150R mutation was not necessary to potentiate the acquisition of 151 variants, as a virus with H150 still acquired D151G populations, when coupled with the Sydney/2014 HA (Fig. 3). Interestingly, the HA of Sydney/2014 was able to drive the selection of NA 148/151 variants in the NA of Sydney/2014 (Fig. 2) whereas the older HA of Wisconsin/2005 was not (Fig. 3). This difference in the ability of HAs from different eras to select NA mutations shows that it is the declining ability of the HA of A/H3N2 viruses to bind cellular receptors in tissue culture that drives the selection of NA variants to assist with receptor binding.

Xue *et al*. [5] demonstrated that virus populations with a 50 : 50 mixture of the D151 and G151 genotype had a growth advantage in MDCK-SIAT1 cells over viruses with either genotype alone. However, for Sydney/2014 such cooperation was not observed in MDCK-SIAT1 cells but did occur in MDCK cell culture (Fig. 4). The 2007 virus used by Xue *et al*. [5] found an equilibrium at approximately 50 : 50 D151 : G151 after serial passage experiments in MDCK-SIAT1 cells, suggesting that this was the optimal ratio to achieve a balance between HA and NA functions in this cell line. Subsequent infection with a 50 : 50 mixture of variants of this virus verified a cooperative effect in MDCK-SIAT1 cells. In contrast, Sydney/2014 selected for a population skewed towards a D151 genotype during serial passage in MDCK-SIAT1 cells (Fig. 2). This suggests that the 50 : 50 mixture of genotypes used in Fig. 4 was sub-optimal in terms of the HA : NA functional balance preferred by this virus in MDCK-SIAT1 cells and explains why complementation was not observed. In MDCK cells, passage of Sydney/2014 selected for a mixture closer to 50 : 50 D151 : G151 (Fig. 2) and cooperation was observed at this ratio in this cell line (Fig. 4). These findings highlight that HAs with subtly different binding properties will likely select for different D151 : G151 NA ratios and that this balance will also depend on cell type due to differences in receptor types between lines. The need for a balance between the functions of HA and NA is essential for influenza virus fitness and perturbations to this balance are rapidly redressed by mutations to modulate the binding function of HA and/or the enzymatic activity, stalk length or efficiency of cell surface trafficking of NA [15–20]. While under the selection pressure of MDCK cell culture, the acquisition of a mixed genotype at NA 148/151, which confers receptor binding while maintaining some sialidase function, is another example of a mechanism by which influenza viruses may find a functional balance between HA and NA.

Here, in an attempt to return cell-passaged H3N2 isolates to a clinical genotype that lacks NA 148/151 receptor-binding variants, a panel of seven isolates was passaged in HAE cells. Genotypes associated with NA-mediated receptor binding were reduced in all three HAE-passage replicates for six of the eight H3N2 isolates tested and entirely removed in two of these six (Fig. 5). Interestingly, it was the isolates previously used as prototype strains for A/H3N2 vaccines, A/Hong Kong/4801/2014 and A/Switzerland/9715293/2013, whose variants at NA 148 were maintained through HAE passage (Fig. 7). This may be a result of the properties of the HAs of these viruses. For the other isolates, seven out of eight HAE passage replicates that were free of NA 148/151 variants remained free of these variants after a subsequent passage in MDCK-SIAT1 cells (Fig. 7). Infection in the presence of mucus did not result in more selection against NA-mediated binding variants for Sydney/2014 (Fig. 5) or a significant difference in titre between the isolates harvested from the two culture conditions. This finding suggests that in this experimental model of airway infection through mucus, the mucus layer did not significantly inhibit phenotypically mixed particles from accessing the cell surface. Phenotypically mixed virions express both D151 sialidase and G151 binding NA molecules on their surface and therefore would still have some ability to cut through mucins in the mucus barrier and enter cells. This allows the delivery of D151 or G151 genomes to generate single phenotype particles, or in the case of co-infection of a cell, both genomes to generate more phenotypically mixed particles carrying either D151 or G151 genomes. However, the reduced frequency of NA-mediated binding variants in the majority of replicates showed that HAE cells exerted negative selection pressure in multi-round replication and demonstrated the utility of these cells in returning A/H3N2 isolates to a more clinical genotype.

Recently, Takada *et al*. (2019) developed a cell line with greatly reduced expression of α-2,3-linked sialic acid and overexpression of α-2,6-linked sialic acid to more closely mimic the receptor profile of the human upper airway. They demonstrated greater A/H3N2 isolation rates than in MDCK or α-2,6-linked sialic acid-overexpressing AX4 cells. Promisingly, these cells did not select for NA148/151 variants or mutations at HA158 that confer the loss of an antigenically significant glycosylation in the three isolates tested [21]. However, since an HA mutation was selected in one of the isolates, challenges still remain in developing cell lines that faithfully maintain the sequences of A/H3N2 clinical samples during virus propagation.

Influenza viruses are isolated in National Influenza Centres around the world that may not have access to MDCK-SIAT or newly modified cell lines designed to reduce the acquisition of cell-passage artefacts. The method presented here of passaging isolates back through HAE cells to return them to a clinical genotype can help to increase the number of isolates available for serological testing. This strategy would improve surveillance coverage from geographical regions that lack the cell lines needed to preserve clinical sequences and prevent the acquisition of NA-mediated binding variants.

## Methods

### Cell lines

MDCK, MDCK-SIAT1 and Vero cells were subcultured every 3–4 days in minimal essential medium (MEM) (Sigma) supplemented with 10 % (v/v) foetal calf serum (FCS), 1.5 % HEPES (Sigma) and 1 % l-glutamine (Sigma). For MDCK-SIAT1 cells, maintenance of the plasmid conferring increased α-2,6-sialylation was achieved by including 10 µg ml^−1^ G418 geneticin antibiotic (Gibco) in the growth medium. Cells were incubated at 37 °C, 5 % CO_2_. Transwells containing Mucilair HAE cells (Epithelix) were transferred to a fresh 24-well plate containing 700 µl Mucilair cell culture medium (Epithelix) every 2–3 days using sterile forceps.

### Virus serial passage and growth curves

MDCK or MDCK-SIAT1 cells were cultured to 90 % confluence in six-well flat-bottom plates. Cell medium was aspirated and cells washed once with infection medium [MEM, 1.5 % HEPES, 1 % l-glutamine, 0.0245 % BA, 2.5 ug ml^−1^ TPCK trypsin (Worthington)]. Influenza virus was then diluted in infection medium and 500 µl of inoculum was added to the cell sheet and incubated at RT for 45 min. For growth curves, triplicate wells were infected for harvest at each time point. Inoculum was then aspirated, cells were washed and 3 ml of infection medium was added per well. Plates were incubated at 35 °C, 5 % CO_2_. Supernatant containing virus was harvested at 72 h for serial passage or at intervening time points for growth curves, clarified by centrifugation (600 ***g***, 10 min) to remove cells debris and stored at −80 °C before being thawed for titration and sequencing.

### HAE cell infection

HAE transwells were transferred to a well of a fresh 24-well plate and washed in phosphate-buffered saline (PBS) before apical infection with virus diluted in Mucilair cell culture medium to a final volume of 200 µl before being incubated at 35 °C, 5 % CO_2_ for 45 min. Some transwells were not washed for experimental reasons. The inoculum was then removed and the cells were incubated at 35 °C, 5 % CO_2_ until harvesting at 12, 24, 48 or 72 h time points for extended periods for virus passage experiments. Virus harvests were carried out by apically adding 300 µl of MEM and incubating at 37 °C, 5 % CO_2_ for 30 min before removing the medium and returning the cells to the incubator. Harvests were stored at −80 °C.

### Virus titration

Haemagglutination assays were carried out on virus isolates using 0.7 % guinea pig RBCs in PBS (0.1 % BSA, with or without 40 nM oseltamivir carboxylate) in 96-well V-bottom plates. Plates were incubated for 1 h at RT before haemagglutination was determined visually.

Plaque assays were performed by inoculating 200 µl of log dilutions of virus onto 90 % confluent monolayers of MDCK-SIAT1 cells. Inoculum was incubated at 35 °C, 5 % CO_2_ for 45 min before being aspirated and replaced with overlay medium (MEM, 0.5 % BA, 2 % sodium bicarbonate, 1 % DEAE-dextran, 1 % penicillin/streptomycin, 1 % amphotericin, 2 % l-glutamine, 2.5 ug ml^−1^ TPCK trypsin) mixed with an equal volume of 2 % bactoagarose. Once plugs had set, the plates were incubated inverted at 35 °C, 5 % CO_2_ for 72 h. Bactoagarose plugs were removed, cells stained with naphthalene black and plaques were quantified.

### Virus reverse genetics

The HA and NA genes of Sydney/2014 were extracted and the NA gene modified by the cloning and recombinant PCR techniques described in Nicolson *et al*. [22] to carry a GGT codon for glycine at position 151. A 12-plasmid rescue system in Vero cells was used to generate isogenic viruses with the HA and D151 or G151 NA of Sydney/2014 together with the 6 internal genes of A/Puerto Rico/8/34 (H1N1) as described by Nicolson *et al*. [22].

### Sequencing

Sanger sequencing of PCR products or gene-containing plasmids was carried out by Eurofins Genomics. For NGS, the one-step RT-PCR method of Zhou *et al*. [23] was used to generate PCR products for all eight influenza gene segments. RT-PCR reactions were performed in triplicate for each viral RNA sample. DNA library preparation was carried out using the Nextera XT protocol (Illumina) according to the manufacturer’s instructions and samples were run on an Illumina MiSeq platform at the National Institute for Biological Standards and Control (NIBSC). Downstream processing of .fastq.gz files at the NIBSC was performed using R and Geneious 10.0 software. Data processing at Colindale was performed with in-house protocols on Galaxy. The GISAID accession numbers of sequences derived from clinical samples containing A/H3N2 influenza viruses are as follows: EPI_ISL_168782; EPI_ISL_186565 to EPI_ISL_186616; EPI_ISL_188738 to EPI_ISL_188863; EPI_ISL_192193 to EPI_ISL_192263; EPI_ISL_240905.
